# Association of dengue virus‐specific polyfunctional T‐cell responses with clinical disease severity in acute dengue infection

**DOI:** 10.1002/iid3.271

**Published:** 2019-09-30

**Authors:** Dulharie T. Wijeratne, Samitha Fernando, Laksiri Gomes, Chandima Jeewandara, Geethal Jayarathna, Yashoda Perera, Samurdhi Wickramanayake, Ananda Wijewickrama, Graham S. Ogg, Gathsaurie N. Malavige

**Affiliations:** ^1^ Centre for Dengue Research, Faculty of Medical Sciences University of Sri Jayewardenepura Nugegoda Sri Lanka; ^2^ Infectious Diseases Hospital Angoda Sri Lanka; ^3^ MRC Human Immunology Unit, Weatherall Institute of Molecular Medicine Oxford NIHR Biomedical Research Centre and University of Oxford Oxford UK

**Keywords:** clinical disease severity, cytokines, dengue, polyfunctional T cells

## Abstract

**Introduction:**

Although the role of dengue virus (DENV)‐specific T cells in the pathogenesis of acute dengue infection is emerging, the functionality of virus‐specific T cells associated with milder clinical disease has not been well studied. We sought to investigate how the functionality of DENV–NS3 and DENV–NS5 protein‐specific T cells differ in patients with dengue fever (DF) and dengue hemorrhagic fever (DHF).

**Methods:**

Using intracellular cytokine assays, we assessed the production of interferon γ (IFNγ), tumor necrosis factor‐α (TNF‐α), macrophage inflammatory protein‐1β (MIP‐1β), and CD107a expression in adult patients with acute DF (n = 21) and DHF (n = 22).

**Results:**

Quadruple cytokine‐producing, polyfunctional DENV–NS3‐ and DENV–NS5‐specific T cells were more frequent in those with DF when compared to those with DHF. While DENV–NS3‐ and DENV–NS5‐specific T cells in patients with DF expressed IFNγ > TNF‐α > MIP‐β > CD107a, T cells of those with DHF predominantly expressed CD107a > MIP‐1β > IFNγ > TNF‐α. Overall production of IFNγ or TNF‐α by DENV–NS3‐ and DENV–NS5‐specific T cells was significantly higher in patients with DF. The majority of NS3‐specific T cells in patients with DF (78.6%) and DHF (68.9%) were single‐cytokine producers; 76.6% of DENV–NS5‐specific T cells in those with DF and 77.1% of those with DHF, produced only a single cytokine. However, no significant association was found with polyfunctional T‐cell responses and the degree of viraemia.

**Conclusions:**

Our results suggest that the functional phenotype of DENV‐specific T cells are likely to associate with clinical disease severity.

## 1 INTRODUCTION

Dengue viral infections affect approximately 390 million individuals annually, of which 96 million infections manifest as apparent dengue infections.^1^ Those who have an apparent dengue infection have symptoms ranging from mild dengue fever (DF) to dengue hemorrhagic fever (DHF). DHF is the severe form of disease which is characterized by plasma leakage resulting in pleural effusions, ascites, and shock along with risk of thrombocytopenia, bleeding, and organ dysfunction.^2^ Currently, there is no specific treatment for dengue and careful monitoring of patients for development of complications and fluid management is the only management strategy available. The only licensed dengue vaccine was found to have low efficacy for some serotypes and has been cautioned for the ability to cause severe disease in dengue‐naïve individuals.^3^, ^4^ This is despite this vaccine inducing high levels of neutralizing antibodies^5^ and dengue virus (DENV)‐specific T‐cell responses of similar breadth and magnitude to those seen following natural infection.^6^ Therefore, it appears that that we need to further understand the constituents of a protective immune responses for development of safer and effective dengue vaccines.

Although there is still some controversy regarding the role of T cells in the pathogenesis of dengue infection, more recent studies have shown that DENV‐specific T cells are likely to have a protective role.^7^, ^8^, ^9^, ^10^ Recently, we reported that patients with DF had significantly higher DENV‐specific T‐cell responses than those with more severe forms of illness, which appeared very early in illness.^11^ Moreover, we also found that the magnitude of the DENV‐specific T‐cell response inversely correlated with the degree of viraemia, further suggesting that T cells have a protective role in acute dengue.^11^

Apart from the breadth and magnitude of a T‐cell response, their functionality has also been shown to be crucial in protection against many infections.^12^, ^13^, ^14^ Polyfunctional T cells that produce multiple types of cytokines have been shown to be protective and correlate with resolution of viraemia, especially in chronic viral infections.^15^, ^16^, ^17^ Polyfunctional T‐cell responses have shown to correlate with the degree of protection with vaccines, rather than pathogen‐specific T cells inducing interferon γ (IFNγ) responses alone.^18^ Polyfunctional virus‐specific T cells have also shown to be important in prevention of virus reactivation in infections such as cytomegalovirus.^12^

It was shown that in individuals who were naturally infected with the DENV, polyfunctional CD8+ and CD4+ T‐cell responses of higher magnitude and breadth were seen for the human leukocyte antigen (HLA) alleles associated with protection.^7^, ^9^ We observed that although individuals who were naturally infected with the DENV resulting in either inapparent or a severe dengue infection had the same magnitude of DENV‐specific T‐cell responses, they differed in functionality. For instance, while DENV‐specific T cells of those who had previous inapparent dengue made higher levels of granzyme B, those with past severe disease made more TNF‐α.^19^ Therefore, as with many other infections, apart from the magnitude and breath of the T‐cell response, the type of cytokines produced by virus‐specific T cells appear to associate with protection or pathogenesis. While many studies have been carried out to elucidate the functionality of T‐cell responses in dengue, these have been limited to studying T‐cell specific for particular HLA types by using tetramers/pentamers.^10^, ^20^ As the functionality of DENV‐specific T cells in acute dengue have not been assessed previously in relation to clinical disease severity and resolution of viraemia, we sought to determine whether polyfunctional CD8+ T‐cell responses to DENV were associated with milder disease and early resolution of viraemia.

## 2 METHODS

### 2.1 Recruitment of patients

We recruited 43 adult patients with acute dengue infection, during a single outbreak from the National Infectious Diseases Institute following informed written consent. Patients were recruited between days 3 and 7 of illness and all clinical features were recorded several times per day, from time of admission to discharge. Ultrasound scans were carried out to identify the presence of fluid leakage in pleural and peritoneal cavities. Full‐blood counts and liver‐transaminase levels were performed serially during the duration of illness. Clinical disease severity was classified as per the 2011 WHO dengue diagnostic criteria.^2^ Accordingly, patients with pleural effusions or ascites or those with a rise in their hematocrits 20 mm Hg or higher of their baseline were classified as having DHF. Shock was defined as the presence of cold clammy skin, in combination with a narrowing pulse pressure of 20 mm Hg or lower. On the basis of this classification, 22 patients had DHF and 21 patients had DF.

### 2.2 Ethics statement

The study was approved by the Ethical Review Committee of The University of Sri Jayewardenepura. All patients were recruited following written consent.

### 2.3 Serology

Acute dengue infection was confirmed in serum samples by polymerase chain reaction (PCR) and dengue antibody identification. Dengue antibody assays were performed using a commercial capture‐immunoglobulin M (IgM) and IgG ELISA (Panbio, Brisbane, Australia).^21^ According to WHO criteria, patients with an IgM:IgG ratio of more than 1.2 were considered under primary dengue infection, while patients with IgM:IgG ratios less than 1.2 were categorized under secondary dengue infection.^2^ The DENV‐specific IgM and IgG enzyme‐linked immunosorbent assay (ELISA) was also performed to determine the semiquantitative DENV‐specific IgM and IgG titers and the results were reported in PanBio units.

### 2.4 Qualitative and quantitative assessment of viral loads

DENV viruses serotyping and quantification was carried out as previously described.^22^ RNA was extracted from the serum samples using QIAamp Viral RNA Mini Kit (Qiagen) as per the manufacturer's protocol. Multiplex quantitative real‐time PCR was performed as previously reported using the CDC real‐time PCR assay to detect DNV. Furthermore, the technique was modified to quantify the DENV. Oligonucleotide primers, a dual‐labeled probe for all DENV 1 to 4 serotypes (Life Technologies, India) were used and this was based on published sequences.^23^ To quantify viruses, standard curves of DENV serotypes were produced as previously reported in Fernando et al.^22^

### 2.5 Peptides

The peptide arrays spanning NS3 (DENV3, Philippines/H87/1956, NS3 protein, NR‐2754) and NS5 proteins (DENV2, New Guinea C, NS5 protein, NR‐2746) from the NIH Biodefense and Emerging Infections Research Resource Repository, NIAID, NIH were used in the study. DENV‐NS3 peptide array is comprised of 105, 14‐ to 17‐mer peptides while NS5 proteins were consisted of 156 peptides. The peptides were reconstituted as previously reported.^24^ DENV‐NS3 and ‐NS5 peptides were pooled separately to represent the DENV‐NS3 and ‐NS5 proteins. Final concentration of dimethyl sulfoxide is less than 0.5%.

### 2.6 Intracellular cytokine assays

The following monoclonal antibodies from Biolegend were used in this study after optimization by serial dilutions: anti‐CD3‐APC Cy7 (clone OKT3), anti‐CD8‐BV650 (clone SK1), anti‐IFN‐γ‐APC (clone 4S. B3), anti‐CD107a‐FITC (clone H4A3), anti‐TNF‐α‐BV605 (clone Mab11). Anti‐MIP1‐β‐PE (clone 24006) was purchased from R&D Systems and LIVE/DEAD Fixable Aqua Dead Cell Stain Kit, for 405 nm excitation was from Thermo Fisher Scientific. Intracellular staining was carried out as previously described.^25^, ^26^ Briefly, to determine CD107a expression, peripheral blood mononuclear cells (PBMCs) were stained with anti‐CD107a‐fluorescein isothiocyanate (FITC) monoclonal antibodies for 30 minutes at 1 to 2 × 10^6^/mL in RPMI 1640 plus 10% fetal calf serum (FCS), before stimulation with the antigen. The PBMCs were then stimulated with either DENV‐NS3 or DENV‐NS5 15‐mer overlapping peptides for 16 hours according to manufacturer's instructions in the presence of Brefeldin A and Monensin (Biolegend) as previously described.^25^, ^27^ The PBMCs were stained with anti‐CD3 and ‐CD8, permeabilized and fixed with Cytofix/Cytoperm (Biolegend) and then stained for intracellular TNF‐α, IFNγ, or MIP‐1β as previously described.^25^, ^26^ LIVE/DEAD Fixable Aqua Dead Cell Stain was used according to the manufacturer's protocol to exclude dead cells.

Samples were acquired using a Guava‐easy Cyte 12 HT Flowcytometer (Merck) and analyzed with FCS Express 6 Flow Research Edition. A hierarchical gating strategy was used to gate live, single, CD3+, and CD8+ T cells (Figures S1 and S2). Each antibody was titrated to determine the optimum concentration to use by comparing it with Fluorescence Minus One (FMO) controls. Gates for TNF‐α, MIP1‐β, IFN‐γ, and CD107a production in DENV–NS3‐ and DENV–NS5‐stimulated cells were set using the unstimulated controls (Figure S2). After the gates for each effector function were created, the data were analyzed using Boolean combination gating to test 15 response patterns for the four functions tested as previously described.^28^ Unstimulated controls were used in all assays.

### 2.7 Statistical analysis

Statistical analysis was carried out using PRISM version 6. As the data were not normally distributed, differences in means were compared using the Mann‐Whitney *U* test (two‐tailed). The Spearman rank‐order correlation coefficient was used to evaluate the correlation between variables. The differences in the frequency the various combinations of single, double, and triple cytokine‐producing DENV‐specific T cells in patients with DF and DHF were analyzed using the Holm‐Sidak method. The relative proportion of single, double, triple, and quadruple cytokine‐producing DENV–NS3‐ and DENV–NS5‐specific T cells in patients with DF and DHF, was calculated as previously described.^28^ The differences between the single, double, triple, and quadruple cytokine‐producing T cells in patients with DF and DHF was analyzed using the Mann‐Whitney *U* test (two‐tailed).

## 3 RESULTS

### 3.1 Clinical and laboratory characteristics of patients

The clinical and laboratory features of the patients are shown in Table 1. In summary, 22 of 43 patients had DHF and 21 had DF. Nineteen of 22 (86.4%) DHF patients displayed ascites and 10 of 22 (45.4%) had pleural effusions. None of the patients developed shock and only one of 43 patients developed bleeding manifestations. The median duration of illness when recruited to the study for patients with DF was 5, (IQR, 4‐5) days and for patients with DHF was 6 (IQR, 5‐7) days.

**Table 1:** Clinical and laboratory features of patients with DF and DHF

Clinical findings	DHF (n = 22)	DF (n = 21)
	N (%)	N (%)
Vomiting	5 (22.7)	5 (23.8)
Abdominal pain	12 (54.5)	9 (42.9)
Hepatomegaly	4 (18.2)	0
Bleeding manifestations	1 (4.5)	0
Pleural effusion	10 (45.5)	0
Ascites	19 (86.4)	0
Lowest platelet count, cells/mm^3^		
<20 000	11 (52.4)	0
20 000‐50 000	7 (33.3)	1 (4.8)
50 000‐100 000	2 (9.5)	9 (42.9)
>100 000	1 (4.5)	11 (52.4)
Lowest lymphocyte count		
<750	2 (9.5)	3 (14.3)
750–1500	8 (38.1)	10 (47.6)
>1500	11 (52.4)	8 (38.1)
Infecting serotype		
DENV1	0	0
DENV2	19 (90.5)	16 (76.2)
DENV3	0	0
DENV4	0	0
Aviraemia	2 (9.5)	5 (23.8)
Inconclusive	0	1 (4.7)

Abbreviations: DENV, dengue virus; DF, dengue fever; DHF, dengue hemorrhagic fever.

### 3.2 Frequency of production of different cytokines by DENV‐specific T‐cell responses in acute dengue

During an acute secondary dengue infection, the quality and quantity of memory T‐cell responses to the previously infecting DENV are likely to shape the immune responses in subsequent dengue infections. To assess the functionality of the DENV‐specific T‐cell response in relation to clinical disease severity, we initially assessed their ability to produce different cytokines. The responses to DENV‐NS3 and ‐NS5 pool of 15‐mer peptides were investigated as T‐cell responses in acute dengue, as these have shown to be the two main proteins inducing CD8+ T‐cell responses.^7^, ^29^, ^30^

**Figure 1:**
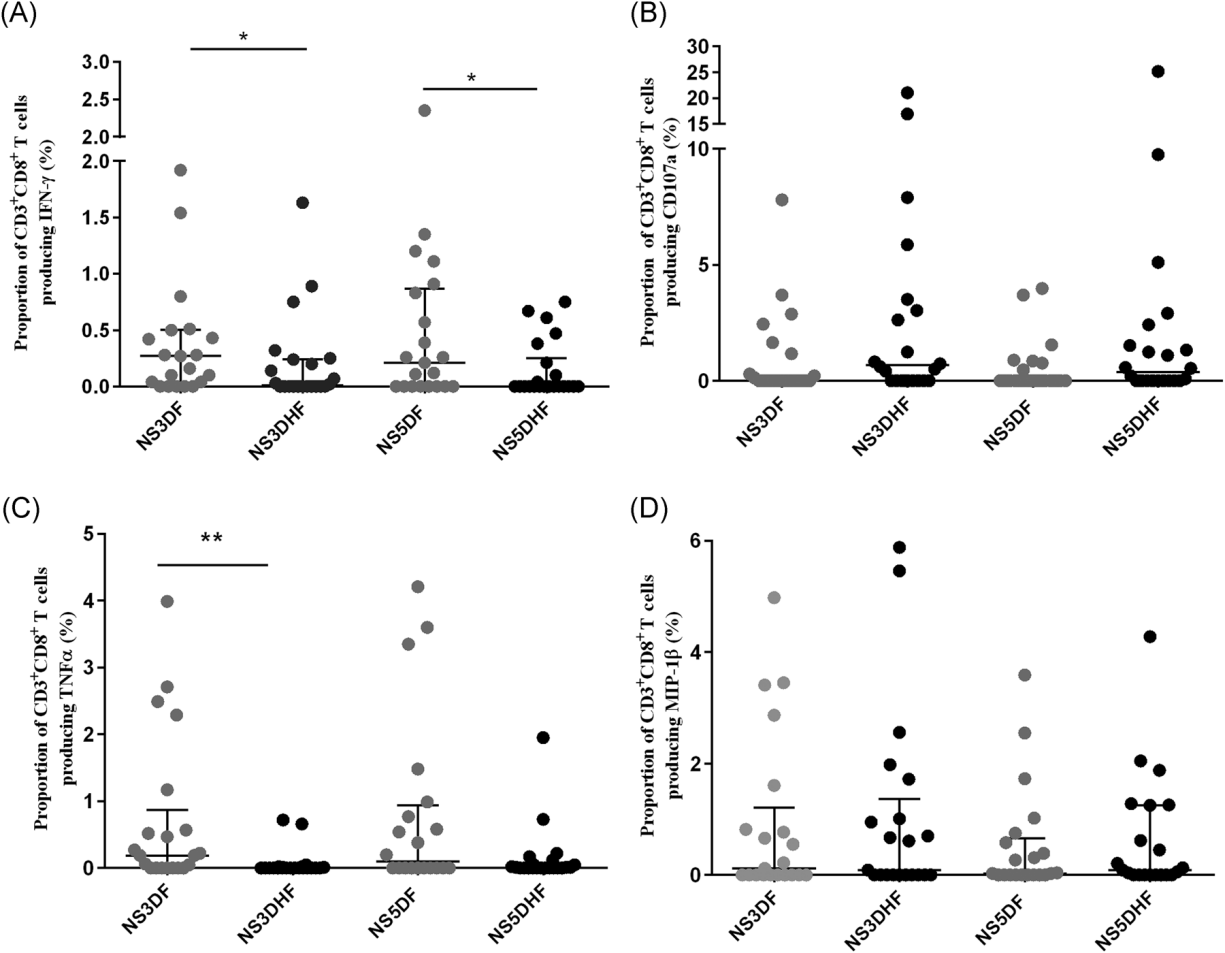
Overall production of cytokines by DENV–NS3‐ and DENV–NS5‐specific T cells in patients with DF and DHF. IFNγ (A), CD107a (B), TNF‐α (C), and MIP‐1β (D) was measured by surface and intracellular cytokine staining in patients with DF (n = 21) and DHF (n = 22). The error bars indicate the median and the IQR. DENV, dengue virus; DF, dengue fever; DHF, dengue hemorrhagic fever; IFNγ, interferon γ; IQR, interquartile range; MIP‐1β, macrophage inflammatory protein‐1β; TNF‐α, tumor necrosis factor‐α. *P < .05 and **P < .001

DENV–NS3‐specific CD8+ T cells of patients with DF produced significantly more (*P* = .045) IFNγ (median = 0.28; IQR, 0%‐0.51%) compared to those of patients with DHF (median, 0; IQR, 0%‐0.24%) (Figure 1A). DENV–NS5‐specific CD8 + T cells of patients with DF also produced significantly more (*P* = .046) IFNγ (median, 0.21; IQR, 0%‐0.87%) compared to those with DHF (median, 0; IQR 0%‐0.28%) (Figure 1A). Although the frequency of TNF‐α producing DENV–NS3‐specific CD8+ T cells were significantly higher (*P* = .003) in patients with DF (median, 0.2; IQR, 0%‐0.87%) compared to patients with DHF (median, 0; IQR, 0%‐0%) (Figure 1C) there was no significant difference seen for DENV–NS5‐specific CD8+ T cells between DF and DHF patients (*P* = .17). In contrast, although not significant, CD107a expression was higher in patients with DHF for both DENV–NS3 (median, 0.61; IQR, 0%‐3.5% of CD8+ T cells) and DENV–NS5‐specific T‐cell responses (median, 0.32; IQR, 0%‐1.48% of CD8+ T cells) compared to patients with DF who exhibited lower CD107a expression following DENV–NS3 (median, 0; IQR, 0%‐1.14% of CD8+ T cells) and DENV‐NS5 stimulation (median, 0; IQR, 0%‐0.69% of CD8+ T cells) (Figure 1B). Ten of 21 (47.6%) of those with DF and 13 of 22 (59.1%) of those with DHF upregulated CD107a in response to NS3. Only two patients with DF had expression rates of greater than 3% whereas seven patients with DHF had rates over 3%. When stimulated with NS5, 7 of 21 (33.3%) of those with DF expressed CD107a compared to 13 of 22 (59.1%) of those with DHF. However, there did not appear to be a difference in MIP‐1β production in patients with DHF for both DENV‐NS3 (median, 0.09; IQR, 0%‐1.3% of CD8+ T cells) and ‐NS5 (median, 0.09; IQR, 0%‐1.25% of CD8+ T cells) compared to those with DF (for NS3, median, 0.11; IQR, 0%‐1.23% and for NS5, median, 0; IQR, 0%‐0.65% of CD8+ T cells) (Figure 1D).

On the basis of the IgM and IgG ratios, 35 of 43 patients could be classified as having either primary or secondary dengue, based on the WHO guidelines.^2^ Of the 35 patients, 17 had a primary dengue infection and 18 had a secondary dengue infection. There was no difference in production of cytokines (IFNγ, TNF‐α, and MIP‐β) in those with primary dengue compared to secondary dengue, when stimulated with either NS3 or NS5. However, although not significant, there was a trend towards increase in CD107a expression in patients with secondary dengue compared to those with primary dengue for both NS3 and NS5.

### 3.3 Association of DENV‐specific polyfunctional CD8+ T cells and disease severity

**Figure 2:**
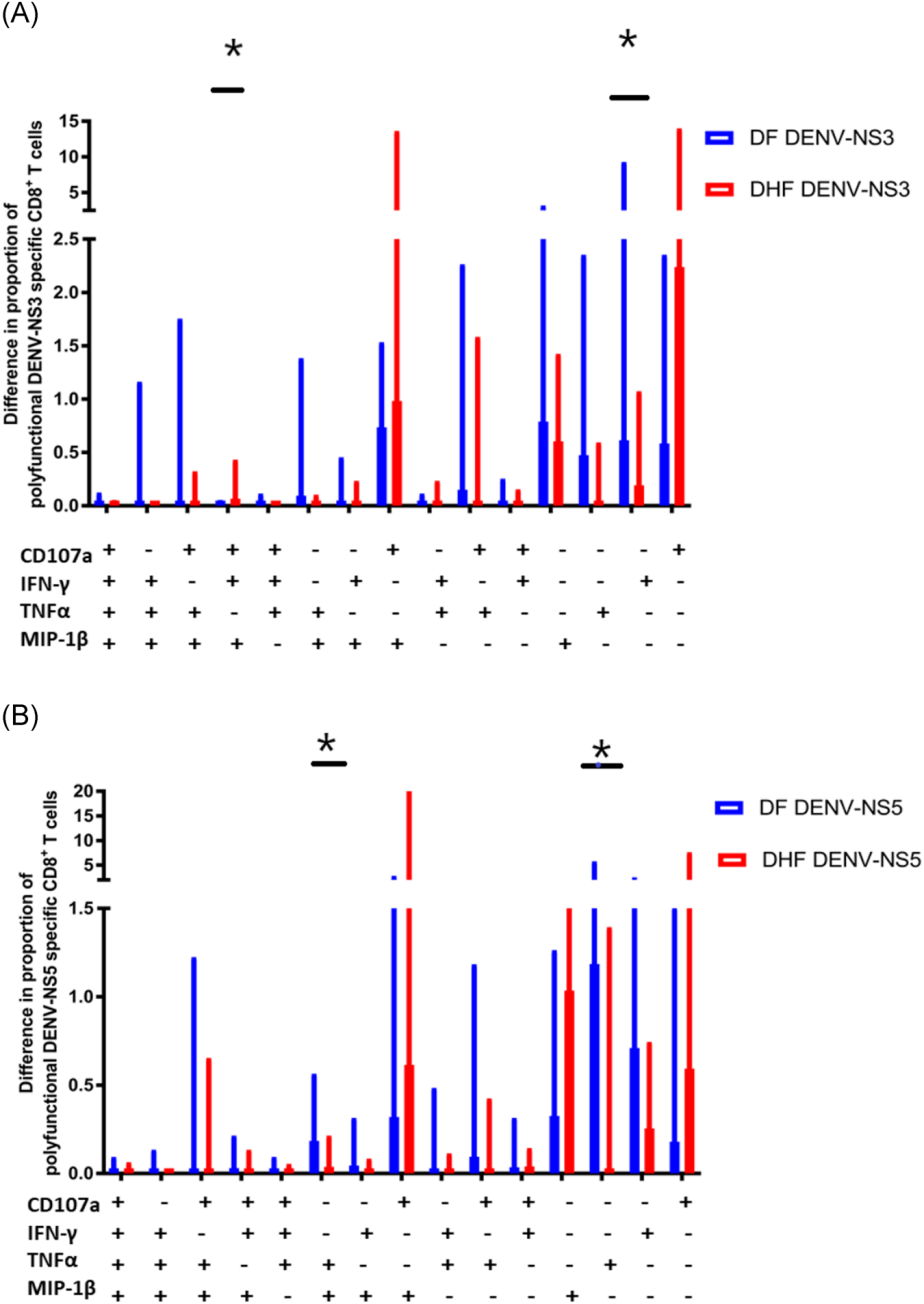
Frequency of single, double, triple, and quadruple cytokine‐producing DENV–NS3‐ and DENV–NS5‐specific T cells in patients with DF and DHF. Fifteen combinations of cytokine‐producing T cells were assessed by intracellular cytokine staining in patients with DF (n = 21) and DHF (n = 22) by using the Boolean combination gates. The error bars indicate the median and the IQR. DENV, dengue virus; DF, dengue fever; DHF, dengue hemorrhagic fever; IFNγ, interferon γ; IQR, interquartile range; MIP‐1β, macrophage inflammatory protein‐1β; TNF‐α, tumor necrosis factor‐α. *P < .05

To assess the polyfunctionality of DNV‐responsive T cells we assessed the ability of T cells to produce multiple cytokines or their ability to carry out certain effector functions by determining the frequency of 15 different combinations of IFNγ, TNF‐α, CD107a, and MIP‐1β production by DENV–NS3‐ (Figure 2A) and DENV–NS5 (Figure 2B)‐specific CD8+ T cells in patients with DF and DHF. To correct for multiple corrections, the data were analyzed using the Holm‐Sidak method. While DENV–NS3‐specific T cells that produced a combination of CD107a/IFNγ/MIP‐1β were significantly higher in patients with DHF (*P* = .049), DENV–NS3‐specific T cells that produced TNF‐α alone was significantly higher (*P* = .02) in patients with DF (Figure 2A). DENV–NS5‐specific T cells which produced a combination of TNF‐α/MIP‐1β and those which produced TNF‐α alone were significantly higher in those with DF (*P* = .03 and *P* = .02, respectively; Figure 2B).

Overall, in patients with DF, DENV–NS3‐specific T cells producing IFNγ alone (median, 0.21; 0%‐0.61% of CD8+ T cells) were more frequent than those producing TNF‐α (median, 0.1; IQR, 0%‐0.45% of CD8+ T cells). The frequency of DENV–NS3‐specific T cells in patients with DF were as follows: IFNγ > MIP‐1β > TNF‐α > CD107a > CD107a/MIP‐1β > CD107a/TNF‐α > CD107a/TNF‐α/MIP‐1β producing cells. The frequency DENV–NS3‐specific T cells in patients with DHF were as follows: CD107a > CD107a/MIP‐1β > MIP‐1β > IFNγ. The frequency of DENV–NS5‐specific T cells in patients with DF were as follows: TNF‐α > IFNγ > CD107a/MIP‐1β > MIP‐1β > CD107a > TNF‐α/MIP‐1β. The frequency of DENV–NS5‐specific T cells in patients with DHF were as follows: CD107a/MIP‐1β > CD107a > IFNγ > MIP‐1β. Therefore, the cytokine signature of DENV–NS3‐ and DENV–NS5‐specific T cells appear to be very different in those with DF compared to those with DHF, with the DENV–NS3‐ and DENV–NS5‐specific T cells predominantly expressing CD107a and MIP‐1β and those with DF predominantly producing IFNγ and TNF‐α.

### 3.4 The association between number of cytokine‐producing T cells and clinical disease severity

**Figure 3:**
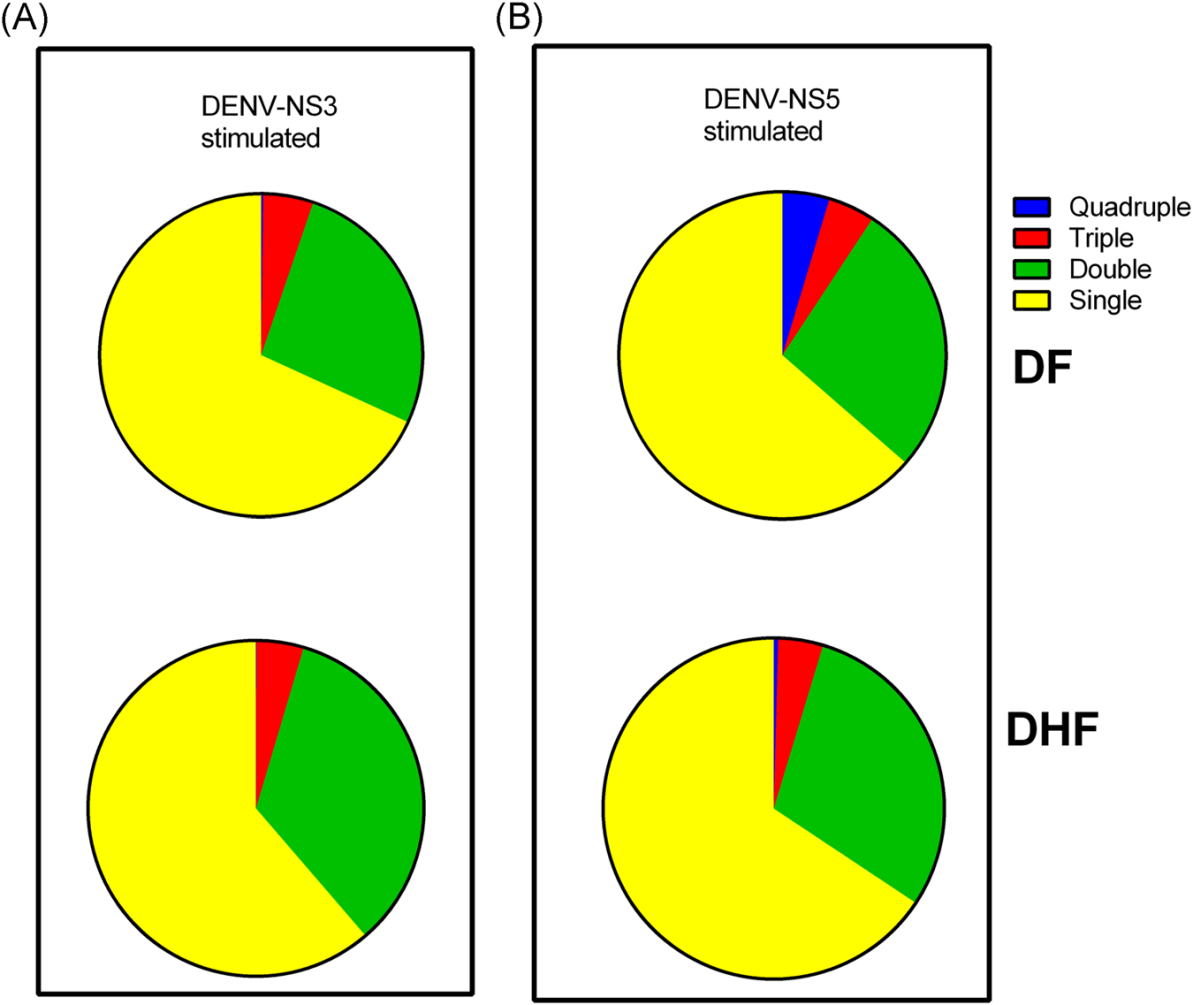
Proportions of polyfunctional T cells in patients with DF and DHF. The proportion of single, double, triple, and quadruple cytokine‐producing (A) DENV–NS3‐ and (B) NS5‐specific T cells were compared in patients with DF (n = 21) and DHF (n = 22). DENV, dengue virus; DF, dengue fever; DHF, dengue hemorrhagic fever

Next, we compared quality of the DENV‐specific T‐cell responses in patients with DF and DHF by calculating the frequency of single, double, triple, and quadruple effector DENV–NS3‐ and DENV–NS5‐specific CD8+ T‐cell proportions regardless of cytokine/effector function combination within the DENV‐specific cytokine‐producing CD8+ T‐cell population (Figure 3). We did not find any significant differences between single, double, triple, and quadruple DENV–NS3‐ and DENV–NS5‐specific CD8+ T‐cell proportions within the DENV‐specific cytokine‐producing CD8+ T‐cell population in patients with DF and DHF (*P* > .05).

0.2% NS3‐specific T cells in patients with DF were quadruple cytokine/effector producers (IFNγ, TNF‐α, CD107a, and MIP1β) while only 0.04% of DENV–NS3‐specific T cells in patients with DHF expressed all four effectors. Furthermore 4.6% of DENV–NS5‐specific T cells were quadruple cytokine/effector producers in patients with DF and only 0.4% in patients with DHF. Therefore, DENV–NS3‐ and NS5‐specific T cells of those with DF were more likely to be polyfunctional. The majority of NS3‐specific T cells in patients with DF (78.6%) and DHF (68.9%) were single cytokine/effector producers. A similar trend was seen for responses to NS5, with 76.6% of those with DF and 77.1% of those with DHF, producing only a single cytokine/effector. Only 21.4% of NS3‐specific T cells in patients with DF and 23.4% in those with DHF produced two cytokines/effectors and for NS5, only 22.7% of those with DF and 18.1% of those with DHF, produced two.

### 3.5 Relationship between functionality of DENV‐specific T cells and viraemia

In our previous study, we found that IFNγ‐producing DENV–NS3‐specific T cells inversely associated with the degree of viraemia.^11^ However, in this study, we did not observe such a relationship with IFNγ, but instead found that the overall DENV–NS5‐specific MIP‐1β producing CD8+ T cells negatively correlated with the degree of viremia in patients with DF (Spearman *r* = −0.51, *P* = .018), but not in those with DHF. We further analyzed the relationship between polyfunctional T‐cell responses and the degree of viraemia. We only found that in patients with DHF, NS5‐specific T cells expressing a combination of CD107a/MIP‐1β/IFNγ, positively and significantly correlated with the degree of viraemia (Spearman *r* = 0.44, *P* = .046). No other cytokine combination had any association with the degree of viraemia.

## 4 DISCUSSION

In this study, we have shown that quadruple cytokine‐/effector‐expressing, polyfunctional DENV–NS3‐ and DENV–NS5‐specific T cells were more frequent in those with DF when compared to those with DHF. In addition, the hierarchy of cytokine production was significantly different in those with severe and milder forms of dengue. For instance, while in those with DF, DENV–NS3‐, and NS5‐specific T cells expressed IFNγ > TNF‐α > MIP‐β > CD107a, those with DHF were of predominantly CD107a > MIP‐1β > IFNγ > TNF‐α. Overall production of IFNγ or TNF‐α by DENV–NS3‐ and DENV–NS5‐specific T cells was significantly higher in patients with DF. Therefore, the preferential production of TNF‐α and IFNγ, which are two potent antiviral cytokines by CD8+ T cells, compared with its degranulation capacity appeared to associate with milder clinical disease. However, in this study, we only investigated the functionality of CD8+ T‐cell responses in patients with acute DENV2 infection, as this was the only circulating serotype during this time period in Sri Lanka.^31^ Although in our previous study, we did not observe any differences in the frequency of IFNγ producing DENV‐specific T cells in acute DENV1 and DENV2 infection, it would be important to study the polyfunctionality of the DENV‐specific T‐cell responses during infections with different DENV serotypes.

Although the overall frequency of DENV–NS3‐ and DENV–NS5‐specific T cells was low for all cytokines that were evaluated, they were comparable to the frequencies of antigen‐specific T cells seen in the acute phase of other virus infections such as influenza,^32^ measles,^33^ and Ebola.^34^ Therefore, although we observed a low frequency of DENV‐specific CD8+ T‐cell responses, these lower frequencies are likely to be important in controlling the viraemia, as seen with acute viral infections. In our previous studies, we have shown that those with milder forms of dengue have higher frequencies of DENV‐specific T‐cell responses,^11^, ^25^ whereas, the majority of patients with severe disease did not have any detectable virus‐specific T‐cell responses. In this study, DENV‐specific T cells produced significantly higher IFNγ and TNF‐α in those with milder disease, further suggesting that the DENV‐specific T cells appear to associate with milder disease.

There has been some speculation that proinflammatory cytokines produced by DENV‐specific T cells contribute to clinical disease severity by leading to endothelial dysfunction.^20^, ^35^, ^36^ Highly cross‐reactive, DENV‐specific CD8+ T‐cell clones, which produced cytokines such as TNF‐α, were thought to contribute to vascular leak. Indeed, anti‐TNF‐α treatment in dengue mouse models was shown to increase survival in experimental DENV infection.^37^ However, it has been shown that DENV‐specific T‐cell responses were absent or present in very low frequencies when the patient enters the critical phase of illness, and that TNF‐α production by T cells did not associate with severe disease.^20^, ^25^, ^38^, ^39^ Indeed it was shown that children who had a higher frequency of DENV‐specific T cells producing TNF‐α, IFNγ, and interleukin‐2 were more likely to have the development of subclinical infection rather than symptomatic illness.^40^ In human and animal influenza viral infection, TNF‐α was shown to have more potent antiviral effects than IFNγ or IFNα.^41^ Therefore, both TNF‐α and IFNγ production by DENV‐specific T cells appear to be important in reducing disease severity, rather than causing disease pathogenesis. However, neither TNF‐α or IFNγ production by CD8+ T cells correlated with the degree of viraemia. Instead, it was MIP‐1β producing CD8+ T cells in patients with DF, which inversely correlated with the degree of viraemia. Although we previously reported that IFNγ‐producing T cells as determined by ex vivo ELISpot inversely correlated with the viraemia, we believe that we did not see a similar observation, as we only investigated CD8+ T cells in this study compared with the overall DENV–T‐cell responses in our previous study.^11^

In healthy individuals who were naturally exposed to the DENV, the majority of DENV‐specific T cells produced two cytokines, irrespective whether they were simulated with conserved‐ or serotype‐specific peptides.^7^ However, we found that in acute infection only 18.1% to 23.3% of DENV‐specific T cells produced two cytokines/effectors, possibly due to the downregulation of T‐cell receptor signaling pathways involved in cytokine during acute dengue.^38^ Although the mean frequency of single cytokine‐/effector‐producing T cells was between 0.1% and 1.9% of the CD8+ T‐cell population, the mean frequency of T cells producing a combination of TNF‐α/IFNγ, TNF‐α/MIP‐1β, or IFNγ/MIP‐1β was less than 0.3% of all CD8+ T cells. However, CD107a/MIP‐1β expressing CD8+ T cells in patients with DHF was between 1.2% and 1.5% of all CD8+ T cells. The expression of CD107a on the surface of CD8+ T cells has shown to be a reliable marker of cytotoxic ability of these cells.^42^ Although expression of CD107a by CD8+ T cells is thought to associate with protection,^43^ it has shown to cause disease pathogenesis in some infections.^44^ For instance, in cutaneous Leishmaniasis, CD107a expressing cytotoxic T cells were highly active in lesions and in mouse models, it was shown that perforin‐deficient mice, did not develop skin lesions when compared with wild‐type mice.^44^ Furthermore, it was shown that disease progression was not associated with the degree of parasitaemia, but rather with the extent of the presence of cytotoxic T cells in skin lesions.^44^ However, although we found that CD107a expressing or CD107a/MIP‐1β/IFNγ expressing DENV‐specific T cells were higher in patients with DHF, there was no association with the degree of viraemia. Therefore, it appears that CD107a expression might not always be protective and DENV‐specific T cells that are preferentially cytotoxic, as opposed to those producing IFNγ and TNF‐α, associate with disease pathogenesis.

In this study, we used overlapping peptides of the NS3‐DENV3 to assess NS3 responses in patients with an acute DENV2 infection. Although there was some concern that using peptides from different serotypes may induce different responses to other infecting serotypes, due to the similarity in the sequences, many previous studies have used a similar approach.^20^, ^27^, ^45^ In addition, it was also shown that HLA‐A*11‐NS3 epitope‐specific tetramer responses (specific for DENV2) were similar in patients with an acute DENV1 or DENV2 infection.^20^

In summary, we investigated polyfunctional DENV–NS3‐ and DENV–NS5‐specific T‐cell responses in patients with acute dengue and found that DENV‐specific T cells producing quadruple cytokines/effectors were more frequent in patients with DF, and they had a different functional phenotype. While DENV‐specific T cells in those with milder disease preferentially produced IFNγ > TNF‐α > MIP‐1β > CD107a, those with severe disease had a functional phenotype of CD107a > MIP‐1β > IFNγ > TNF‐α. Therefore, our results suggest that not only the magnitude of T‐cell responses, but also the functional phenotype of DENV‐specific T cells associate with disease severity.

## CONFLICT OF INTERESTS

The authors declare that there are no conflict of interests.

## DATA AVAILABILITY

All data is shown within the manuscript, figures, and Supporting Information.

## Supporting information

Supporting information

Supporting information

Supporting information

## References

[1] Bhatt S , Gething PW , Brady OJ , et al. The global distribution and burden of dengue. Nature. 2013;496(7446):504‐507. 10.1038/nature12060 23563266 PMC3651993

[2] WHO SEARO . In: WHO , ed. Comprehensive guidelines for prevention and control of dengue fever and dengue haemorrhagic fever Serious No. 60 New Delhi, India: World Health Organization; 2011:24‐26.

[3] Halstead SB . Safety issues from a Phase 3 clinical trial of a live‐attenuated chimeric yellow fever tetravalent dengue vaccine. Hum Vaccin Immunother. 2018;14(9):2158‐2162. 10.1080/21645515.2018.1445448 29482433 PMC6183135

[4] WHO position paper . Dengue vaccine: WHO position paper, September 2018—Recommendations. Vaccine. 2018;37(35):4848‐4849. 30424888 10.1016/j.vaccine.2018.09.063

[5] Leo YS , Wilder‐Smith A , Archuleta S , et al. Immunogenicity and safety of recombinant tetravalent dengue vaccine (CYD‐TDV) in individuals aged 2‐45 y: phase II randomized controlled trial in Singapore. Hum Vaccin Immunother. 2012;8(9):1259‐1271. 10.4161/hv.21224 22894958 PMC3579907

[6] Weiskopf D , Angelo MA , Bangs DJ , et al. The human CD8+ T cell responses induced by a live attenuated tetravalent dengue vaccine are directed against highly conserved epitopes. J Virol. 2015;89(1):120‐128. 10.1128/JVI.02129-14 25320311 PMC4301095

[7] Weiskopf D , Angelo MA , de Azeredo EL , et al. Comprehensive analysis of dengue virus‐specific responses supports an HLA‐linked protective role for CD8+ T cells. Proc Natl Acad Sci. 2013;110(22):E2046‐E2053. 10.1073/pnas.1305227110 23580623 PMC3670335

[8] Weiskopf D , Cerpas C , Angelo MA , et al. Human CD8+ T‐cell responses against the 4 dengue virus serotypes are associated with distinct patterns of protein targets. J Infect Dis. 2015;212(11):1743‐1751. 10.1093/infdis/jiv289 25980035 PMC4633759

[9] Weiskopf D , Bangs DJ , Sidney J , et al. Dengue virus infection elicits highly polarized CX3CR1+ cytotoxic CD4+ T cells associated with protective immunity. Proc Natl Acad Sci. 2015;112(31):E4256‐E4263. 10.1073/pnas.1505956112 26195744 PMC4534238

[10] Rivino L , Kumaran EA , Thein TL , et al. Virus‐specific T lymphocytes home to the skin during natural dengue infection. Sci Translat Med. 2015;7(278):278ra35‐278ra35. 10.1126/scitranslmed.aaa0526 25761891

[11] Wijeratne DT , Fernando S , Gomes L , et al. Quantification of dengue virus specific T cell responses and correlation with viral load and clinical disease severity in acute dengue infection. PLOS Neglect Tropic Dis. 2018;12(10):e0006540 10.1371/journal.pntd.0006540 PMC6181435 30273352

[12] Choi YJ , Kim SB , Kim JH , et al. Impaired polyfunctionality of CD8(+) T cells in severe sepsis patients with human cytomegalovirus reactivation. Exp Mol Med. 2017;49(9):e382 10.1038/emm.2017.146 28960213 PMC5628278

[13] Yamamoto T , Iwamoto N , Yamamoto H , et al. Polyfunctional CD4+ T‐cell induction in neutralizing antibody‐triggered control of simian immunodeficiency virus infection. J Virol. 2009;83(11):5514‐5524. 10.1128/JVI.00145-09 19297503 PMC2681982

[14] Kasprowicz V , Ward SM , Turner A , et al. Defining the directionality and quality of influenza virus‐specific CD8+ T cell cross‐reactivity in individuals infected with hepatitis C virus. J Clinic Invest. 2008;118(3):1143‐1153. 10.1172/JCI33082 PMC2214846 18246203

[15] Snyder LD , Chan C , Kwon D , et al. Polyfunctional T‐cell signatures to predict protection from cytomegalovirus after lung transplantation. Am J Respir Crit Care Med. 2016;193(1):78‐85. 10.1164/rccm.201504-0733OC 26372850 PMC4731614

[16] Seder RA , Darrah PA , Roederer M . T‐cell quality in memory and protection: implications for vaccine design. Nat Rev Immunol. 2008;8(4):247‐258. 10.1038/nri2274 18323851

[17] Betts MR , Nason MC , West SM , et al. HIV nonprogressors preferentially maintain highly functional HIV‐specific CD8+ T cells. Blood. 2006;107(12):4781‐4789. 10.1182/blood-2005-12-4818 16467198 PMC1895811

[18] Panagioti E , Klenerman P , Lee LN , van der Burg SH , Arens R . Features of effective T cell‐inducing vaccines against chronic viral infections. Front Immunol. 2018;9:276 10.3389/fimmu.2018.00276 29503649 PMC5820320

[19] Jeewandara C , Adikari TN , Gomes L , et al. Functionality of dengue virus specific memory T cell responses in individuals who were hospitalized or who had mild or subclinical dengue infection. PLOS Neglect Tropic Dis. 2015;9(4):e0003673 10.1371/journal.pntd.0003673 PMC4395258 25875020

[20] Mongkolsapaya J , Duangchinda T , Dejnirattisai W , et al. T cell responses in dengue hemorrhagic fever: are cross‐reactive T cells suboptimal? J Immunol. 2006;176(6):3821‐3829. 16517753 10.4049/jimmunol.176.6.3821

[21] Sang CT , Cuzzubbo AJ , Devine PL . Evaluation of a commercial capture enzyme‐linked immunosorbent assay for detection of immunoglobulin M and G antibodies produced during dengue infection. Clin Diagnost Lab Immunol. 1998;5(1):7‐10. 10.1128/cdli.5.1.7-10.1998 PMC121382 9455871

[22] Fernando S , Wijewickrama A , Gomes L , et al. Patterns and causes of liver involvement in acute dengue infection. BMC Infect Dis. 2016;16:319 10.1186/s12879-016-1656-2 27391896 PMC4938910

[23] Santiago GA , Vergne E , Quiles Y , et al. Analytical and clinical performance of the CDC real time RT‐PCR assay for detection and typing of dengue virus. PLOS Neglect Tropic Dis. 2013;7(7):e2311 10.1371/journal.pntd.0002311 PMC3708876 23875046

[24] Malavige GN . Ex vivo ELISpot assay to investigate dengue virus specific T‐cell responses. Methods Mol Biol. 2018;1808:173‐179. 10.1007/978-1-4939-8567-8_15 29956183

[25] Malavige GN , Jeewandara C , Alles KM , et al. Suppression of virus specific immune responses by IL‐10 in acute dengue infection. PLOS Neglect Tropic Dis. 2013;7(9):e2409 10.1371/journal.pntd.0002409 PMC3764236 24040431

[26] Park SH , Shin EC . Direct ex vivo functional analysis of HCV‐specific T cells. Methods Mol Biol. 2019;1911:349‐361. 10.1007/978-1-4939-8976-8_24 30593638

[27] Chu H , George SL , Stinchcomb DT , Osorio JE , Partidos CD . CD8+ T‐cell responses in flavivirus‐naive individuals following immunization with a live‐attenuated tetravalent dengue vaccine candidate. J Infect Dis. 2015;212(10):1618‐1628. 10.1093/infdis/jiv258 25943203 PMC4621245

[28] Ritz N , Strach M , Yau C , et al. A comparative analysis of polyfunctional T cells and secreted cytokines induced by Bacille Calmette‐Guerin immunisation in children and adults. PLOS One. 2012;7(7):e37535 10.1371/journal.pone.0037535 22829867 PMC3400612

[29] Simmons CP , Dong T , Chau NV , et al. Early T‐cell responses to dengue virus epitopes in Vietnamese adults with secondary dengue virus infections. J Virol. 2005;79(9):5665‐5675. 15827181 10.1128/JVI.79.9.5665-5675.2005 PMC1082776

[30] Appanna R , Huat TL , See LL , Tan PL , Vadivelu J , Devi S . Cross‐reactive T‐cell responses to the nonstructural regions of dengue viruses among dengue fever and dengue hemorrhagic fever patients in Malaysia. Clin Vaccin Immunol. 2007;14(8):969‐977. 10.1128/CVI.00069-07 PMC2044482 17567768

[31] Wijewickrama A , Fernando S , Jayerathne GSB , et al. Emergence of a Dengue virus serotype 2 causing the largest ever dengue epidemic in Sri Lanka. bioRxiv. 2018:329318 10.1101/329318

[32] Hoji A , Rinaldo CR Jr. Human CD8+ T cells specific for influenza A virus M1 display broad expression of maturation‐associated phenotypic markers and chemokine receptors. Immunology. 2005;115(2):239‐245. 10.1111/j.1365-2567.2005.02135.x 15885130 PMC1782154

[33] Ota MO , Ndhlovu Z , Oh S , et al. Hemagglutinin protein is a primary target of the measles virus‐specific HLA‐A2‐restricted CD8+ T cell response during measles and after vaccination. J Infect Dis. 2007;195(12):1799‐1807. 10.1086/518006 17492596

[34] Sakabe S , Sullivan BM , Hartnett JN , et al. Analysis of CD8(+) T cell response during the 2013‐2016 Ebola epidemic in West Africa. Proc Natl Acad Sci USA. 2018;115(32):E7578‐E7586. 10.1073/pnas.1806200115 30038008 PMC6094108

[35] Dong T , Moran E , Vinh Chau N , et al. High pro‐inflammatory cytokine secretion and loss of high avidity cross‐reactive cytotoxic T‐cells during the course of secondary dengue virus infection. PLOS One. 2007;2(12):e1192. 18060049 10.1371/journal.pone.0001192 PMC2092391

[36] Mathew A , Rothman AL . Understanding the contribution of cellular immunity to dengue disease pathogenesis. Immunol Rev. 2008;225:300‐313. 18837790 10.1111/j.1600-065X.2008.00678.x

[37] Atrasheuskaya A , Petzelbauer P , Fredeking TM , Ignatyev G . Anti‐TNF antibody treatment reduces mortality in experimental dengue virus infection. FEMS Immunol Med Microbiol. 2003;35(1):33‐42. 10.1111/j.1574-695X.2003.tb00646.x 12589955

[38] Chandele A , Sewatanon J , Gunisetty S , et al. Characterization of human CD8 T cell responses in dengue virus infected patients from India. J Virol. 2016;90(24):11259‐11278. 10.1128/JVI.01424-16 27707928 PMC5126381

[39] Malavige GN , Huang LC , Salimi M , Gomes L , Jayaratne SD , Ogg GS . Cellular and cytokine correlates of severe dengue infection. PLOS One. 2012;7(11):e50387 10.1371/journal.pone.0050387 23209731 PMC3510251

[40] Hatch S , Endy TP , Thomas S , et al. Intracellular cytokine production by dengue virus‐specific T cells correlates with subclinical secondary infection. J Infect Dis. 2011;203(9):1282‐1291. 10.1093/infdis/jir012 21335561 PMC3069729

[41] Seo SH , Webster RG . Tumor necrosis factor alpha exerts powerful anti‐influenza virus effects in lung epithelial cells. J Virol. 2002;76(3):1071‐1076. 11773383 10.1128/JVI.76.3.1071-1076.2002 PMC135862

[42] Aktas E , Kucuksezer UC , Bilgic S , Erten G , Deniz G . Relationship between CD107a expression and cytotoxic activity. Cellular immunology. 2009;254(2):149‐154. 10.1016/j.cellimm.2008.08.007 18835598

[43] Wolint P , Betts MR , Koup RA , Oxenius A . Immediate cytotoxicity but not degranulation distinguishes effector and memory subsets of CD8+ T cells. J Exper Med. 2004;199(7):925‐936. 10.1084/jem.20031799 15051762 PMC2211884

[44] Novais FO , Carvalho LP , Graff JW , et al. Cytotoxic T cells mediate pathology and metastasis in cutaneous leishmaniasis. PLOS Pathogens. 2013;9(7):e1003504 10.1371/journal.ppat.1003504 23874205 PMC3715507

[45] Mongkolsapaya J , Dejnirattisai W , Xu XN , et al. Original antigenic sin and apoptosis in the pathogenesis of dengue hemorrhagic fever. Nat Med. 2003;9(7):921‐927. 12808447 10.1038/nm887

