# Supplementary figures and images for: Association of dengue virus‐specific polyfunctional T‐cell responses with clinical disease severity in acute dengue infection
Source: Immun Inflamm Dis. 2019 Sep 30;7(4):276–85. doi: 10.1002/iid3.271 (PMC6842812; doi:10.1002/iid3.271)

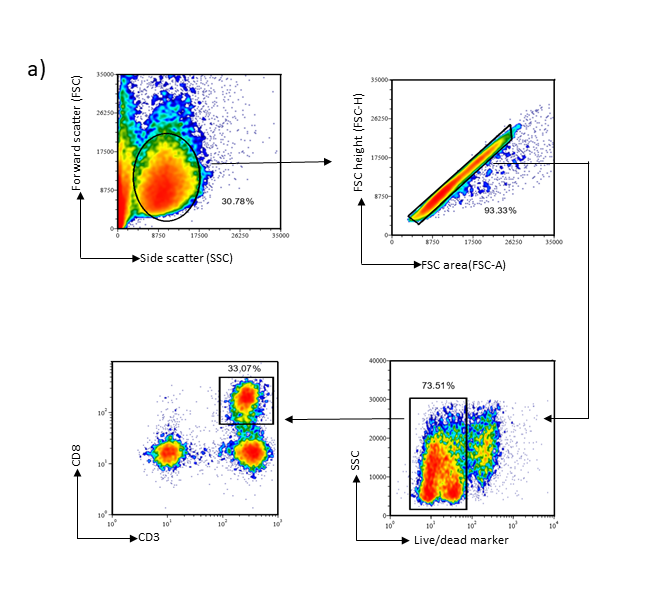

Supplement: Supplementary file 1 — Supporting information [file IID3-7-276-s001.tif]

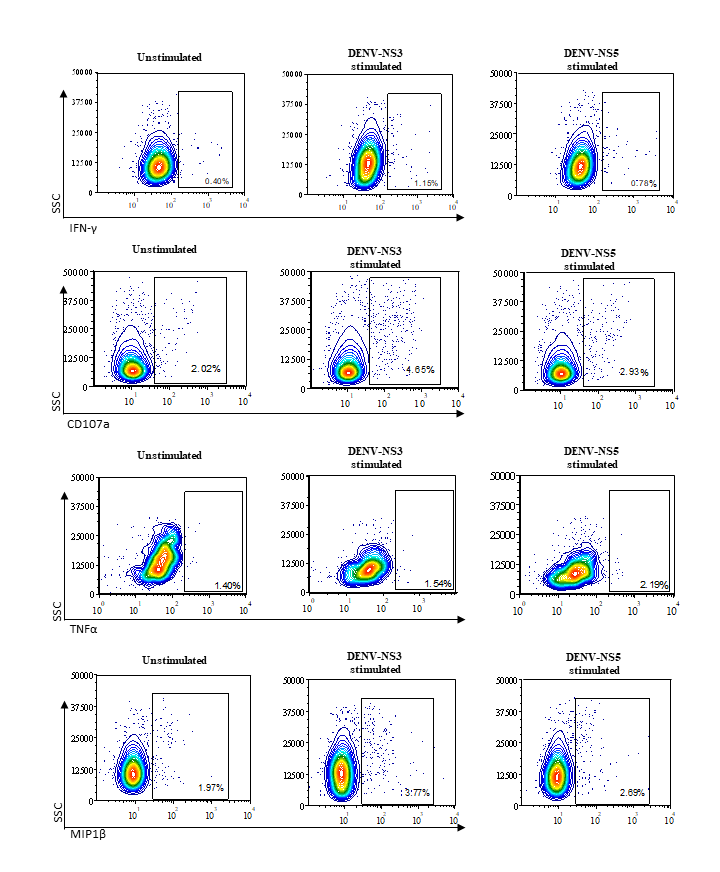

Supplement: Supplementary file 2 — Supporting information [file IID3-7-276-s002.tif]
