# Supplementary material for: Association of dengue virus‐specific polyfunctional T‐cell responses with clinical disease severity in acute dengue infection
Source: Immun Inflamm Dis. 2019 Sep 30;7(4):276–85. doi: 10.1002/iid3.271 (PMC6842812; doi:10.1002/iid3.271)
Supplement: Supplementary file 3 — Supporting information [file IID3-7-276-s003.docx]

**Supplementary figure 1: The hierarchical gating strategy used to gate live, single, CD3+ and CD8+ T cells.** Cells were first gated on the PBMCS, then the singlets were identified by gating on FSC-height and area, these cells were then gated on the live cells and subsequently on CD8+ CD3+ cells.

**Supplementary figure 2: The gating strategy used to define a positive response to CD107a expression and production of IFNγ, MIP-1β and TNFα.** The cells are gated on CD3+CD8+live cells.
